# Synthesis and Physicochemical Stability of a Copaiba Balsam Oil (*Copaifera sp.*) Nanoemulsion and Prospecting of Toxicological Effects on the Nematode *Caenorhabditis elegans*

**DOI:** 10.1021/acsomega.4c05930

**Published:** 2024-09-06

**Authors:** Iverson Conrado Bezerra, Emily Raphaely Souza dos Santos, Jocelin Santa Rita Bisneto, Paloma Paschoal Perruci, Angela Iasmin de Barros Ferreira, Daniel Charles dos Santos Macêdo, Mateus Araújo da Luz, Taynah Pereira Galdino, Giovanna Machado, Nereide Stela Santos Magalhães, Mariane Cajuba de Britto Lira Nogueira, Priscila Gubert

**Affiliations:** † Keizo Asami Institute (iLIKA), Federal University of Pernambuco, Recife 50670-901, Brazil; ‡ Northeast Strategic Technologies Center (CETENE), Recife 50740-545, Brazil; § Department of Pharmaceutical Sciences, Federal University of Pernambuco, Recife 50670-901, Brazil; ∥ Northeast Biomaterials Assessment and Development Laboratory (CERTBIO), Federal University of Campina Grande, Campina Grande 58429-900, Brazil; ⊥ Federal University of Western Bahia (UFOB), Barreiras 47800-000, Brazil

## Abstract

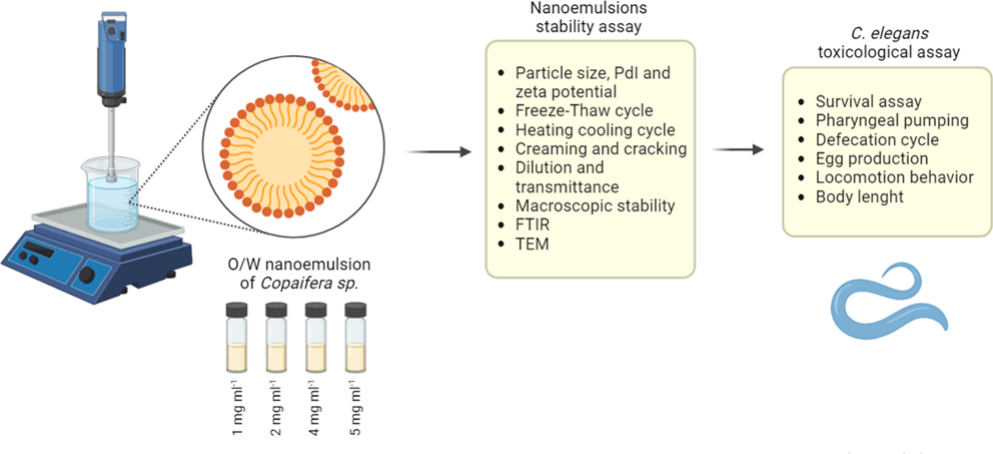

Nanoemulsions are dispersions of oil-in-water (O/W) and water-in-oil (W/O) immiscible liquids. Thus, our main goal was to formulate a nanoemulsion with low surfactant concentrations and outstanding stability using Copaiba balsam oil (Copaifera sp.). The high-energy cavitation homogenization with low Tween 80 levels was employed. Then, electrophoretic and physical mobility properties were assessed, in addition to a one- and two-year physicochemical characterization studies assessment. Copaiba balsam oil and nanoemulsions obtained caryophyllene as a major constituent. The nanoemulsions stored at 4 ± 2 °C exhibited better physical stability. Two years after formulation, the nanoemulsion showed a reduction in the particle size. The size underwent changes in gastric, intestinal, and blood pH, and the PdI was not changed. In FTIR, characteristic bands of sesquiterpenes and overlapping bands were detected. When subjected to freezing and heating cycles, nanoemulsions did not show macroscopic changes in higher concentrations. Nanoemulsions subjected to centrifuge force by 1000 rpm do not show macroscopic instability and phase inversion or destabilization characteristics when diluted. Therefore, the nanoemulsion showed stability for long-term storage. The nematode *Caenorhabditis elegans* was used to assess the potential toxicity of nanoemulsions. The nanoemulsion did not cause toxicity in the animal model, except in the highest concentration tested, which decreased the defecation cycle interval and body length. The toxicity and stability outcomes reinforce the nanoemulsions’ potential for future studies to explore pharmacological mechanisms in superior experimental designs.

## Introduction

Nanoemulsions are oil-in-water (O/W) or water-in-oil (W/O) dispersions of immiscible liquids, being metastable systems generally with the average droplet size being between 20 and 500 nm.^1−3^ Worldwide, nanoemulsions are characterized by different physicochemical methods to evaluate rheology, macroscopic appearance, morphology, viscosity, particle size, surface charge, size distribution, and stability over time. The characterizations provide information about the behavior of nanoemulsions and direct them to different medical uses.^4^

Nanoemulsions have unique applications that make them suitable for the pharmaceutical industry, as they have small particle sizes, high surface area, physical stability, and high bioavailability.^5^ The bioavailability of vitamins and nutraceuticals is increased when applied through nanoemulsions, in addition to promoting increased drug retention time at the target site and enhancing drug release, reducing toxicity, systemic adverse events, and therapeutic dose.^5^

Dissolving nonpolar active compounds is one of the main advantages of using nanoemulsions. Furthermore, they are more advantageous than emulsions and other systems since the particle size favors intravenous, oral, and parenteral administration of medications and ensures controlled release. Furthermore, nanoemulsions guarantee improved transit time, absorption, and efficacy of medications.^6^

Copaiba balsam oil is extracted from trees of the genus Copaifera, subfamily Caesalpinioideae. Around 80% of its molecules are sesquiterpenes, and β-caryophyllene mainly constituting the oleoresin.^7^ It has activities such as antimicrobial,^8^ antifungal,^9^ antioxidant, larvicidal, antiparasitic, and gastroprotective, among others. Furthermore, compounds present in the oil have anti-Alzheimer, anticancer, and anti-inflammatory activity.^10^ β-caryophyllene has demonstrated significant nervous system modulation in preclinical models, effective in several neurodegenerative and inflammatory disorders.^11^

Several nanoemulsions have been synthesized with Copaiba balsam oil. Formulations vary in surfactant, oil viscosity, and high- or low-energy formation method, among others. The synthesis can be realized by using ethanol and high temperatures,^9^ egg lecithin,^12^ triglycerides,^13^ span 80,^13^ or cosurfactants.^14^ However, although stable, the large quantity and diversity of molecules responsible for forming and stabilizing formulations can present high costs, and adverse effects are unknown. Furthermore, heating can generate volatilization of organic compounds with biological activities.

*Caenorhabditis elegans* is a free-living, nonpathogenic nematode with high biological similarity in development and neuronal functions to mammals. Toxicological tests on *C. elegans* are carried out due to their predictive potential in safety assessment. They allow the evaluation of complex toxicological effects due to multiple tissues, making the animal model a bridge between in vitro testing and mammalian tests.^15,16^

Our study proposes the formulation of a low-cost nanoemulsion containing Copaiba balsam oil (*Copaifera sp.*). To our knowledge, this work is the only that used the lowest surfactant levels to produce nanoemulsions of Copaiba balsam oil and evaluates the physicochemical stability for up to two years. Furthermore, we assessed the toxicological effects of the nanoemulsion on the *C. elegans* animal model. Additionally, reducing surfactant levels may be essential for different delivery sites and better assessing toxicity and biological modifications.

## Materials and Methods

### Reagents

Copaiba balsam oil (*Copaifera sp.*) by Sigma-Aldrich Co (St. Louis, MO, EUA) was kindly supplied from Northeast Strategic Technologies Center (Recife, PE, Brazil). Tween 80 was purchased from Synth (Diadema, SP, Brazil). Sodium chloride P.A (Química moderna, SP, Brazil). Hydrochloric acid P.A A.C.S (Anidrol, SP, Brazil). Sodium biphosphate P.A A.C.S (Synth, SP, Brazil). Potassium phosphate monobasic anhydrous P.A (Synth, SP, Brazil). Sodium hydroxide (Synth, SP, Brazil). Methanol (Êxodo científica, SP, Brazil). NGM medium: Agar (EMPROVE, Darmstadt, Germany), Peptone (Sigma aldrich, St. Louis, EUA) and Sodium chloride P.A (Química moderna, SP, Brazil). M9 buffer: Sodium phosphate dibasic P.A A.C.S (Sigma aldrich, St. Louis, EUA), Potassium phosphate bibasic P.A (Synth, SP, Brazil) and Sodium chloride P.A (Química moderna, SP, Brazil). Uranyl acetate (Fisher scientific, EUA), OP50 *Escherichia coli* (Fisher scientific, EUA).

### Chemical Composition of Copaiba Balsam Oil and Nanoemulsion by Gas Chromatography Coupled to Mass Spectrometer (GC-MS)

Gas chromatography–mass spectrometry (GC-MS) analysis was performed on a Clarus 590 gas chromatograph equipped with a PALCOMBI-xt automatic injector, an Agilent VF-1MS column (60 m × 0.32 mm i.d., 1 μm), and a Clarus SQ8S mass spectrometer (PerkinElmer, Waltham, Massachusetts). Helium gas was used as a carrier gas at a flow rate of 1 mL/min. The injector was heated to 250 °C working with a 1:10 split, and 1 μL of the copaiba balsam oil diluted in methanol (2000 ppm) was injected. The oven was programmed as follows: first step: heating gradient at 60–90 °C (for 1 min) at a rate of 7 °C/min; second step: 90–170 °C (for 5 min) at a rate of 3 °C/min; third step: 170–240 °C at a rate of 3 °C p/min. The analysis time was 41.62 min. The detector worked in electron ionization (EI) mode at 70 eV, with an interface temperature of 250 °C (inlet line) and a source (source temp) at 220 °C. Mass fragments were monitored in the range of 50–650 Da. The NIST database from the NIST MS Search Version 2.2 software (National Institute of Standards and Technology, Gaithersburg, MD, USA) was used to identify the compounds. The same conditions were used to evaluate the nanoemulsion of copaiba balsam oil, except the sample concentration was 6000 ppm prepared in methanol.^17^

The compounds present in the copaiba balsam oil were identified by comparing their respective mass spectra with those of other previously analyzed compounds, with the mass spectra of the NIST database (NIST MS Search Version 2.2), and with the chemical composition of copaiba essential oils described in other studies.

### Nanoemulsion Preparation

Oil-in-water (O/W) nanoemulsions were prepared using the high energy emulsification method in the concentrations of 1, 2, 4, and 5 mg mL^–1^ of *Copaifera sp*. balsam oil (Copaiba balsam oil), with the oil-phase volume fraction of 0.1, 0.2, 0.4, and 0.5%, respectively.^18^ The oily phase was composed of copaiba balsam oil without mixing it with other vegetable oils. Tween 80 to 1% (0.05% final concentration in nanoemulsion) in ultrapure water was dissolved in the oily phase. The aqueous phase composed of ultrapure water was added to the mixture with the oily phase and gently stirred by providing a primary formulation. The final homogenization was obtained by the cisalation forces generated by the T25 Ultra-Turrax homogenizer equipped with an S 25 kV-18 G for 15 min (7500 rpm) and exposed to ultrasonication by 10 min with a 1 cm diameter titanium probe, output pulsation 5 at 1s intervals, a 50% active cycle, frequency of 20, 200 W and 40 Hz on the Vibracell 72441 equipment (Bioblock Scientific, USA).^19^ The formulations were stored under different temperature conditions of 4 ± 2, 20 ± 2, and 36 ± 2 °C.

### Particle Size and Polydispersity Index Analysis

The hydrodynamic diameter of the nanoemulsion droplets and polydispersity index (PdI) of the formulations were determined by photon correlation spectroscopy at 25 °C using a Zetasizer Nano ZS (Malvern PCS Instruments, UK). The samples were diluted in ultrapure water type 2 in a ratio of 1:20 (v/v). Analyzes were performed after formulation, 15, 30, 60 days, 1 year, and 2 years after storing the nanoformulations and data obtained by automated analysis with second cumulant adjustment with automatic adjustment ranges based on data quality. The values reported are the mean of three triplicate readings.^20,21^

### Electrophoretic Measurement

The Zeta potential of the nanoemulsions was measured by electrophoretic mobility using a Zetasizer Nano ZS (Malvern PCS Instruments, UK). Readings were carried out at 25 °C, and the samples were diluted at a ratio of 1:20 (v/v) in a saline solution (10 mM NaCl) at pH 6. Analyzes were performed after formulation, 15, 30, 60 days, 1 year, and 2 years after nanoemulsion formulations, and the values reported are the mean of three triplicate readings.^22^

### pH Evaluation

The pH of nanoemulsions was measured by introducing the electrode directly into the samples at 20 ± 2 °C. Analyzes were performed after formulation.^23^

### Viscosity Measurements

The nanoemulsions’ viscosity was measured using a digital viscometer (MVD-20 model, Brazil) with a low-viscosity spindle at 70 rpm at 25 °C. Analyses were performed after formulation preparation, and the values reported are the mean of three triplicate readings.^24^

### Stability of Nanoemulsions at Different pH

Simulated biological fluids (gastric, intestinal, and blood) were performed to evaluate the stability of nanoemulsions. Phosphate buffer solutions (pH 7.4) were made and partly used as a blood pH-simulating solution. In another part of the buffer, a solution of HCL (1 M) was added, obtaining a pH of 1.2 (gastric pH). Monobasic potassium phosphate solutions were prepared, and sodium hydroxide (0.2 M) and ultrapure water (Milli Q, Millipore, USA) were added to obtain a pH 6.8 solution (intestinal pH). In microtubes, 750 μL of pH 1.2, 6.8, and 7.4 solutions were added, and 250 μL of the formulation. The tubes were homogenized by stirring at 25 °C for 1 h. After 1 h, one aliquot was taken to analyze each concentration placed in a quartz cuvette to evaluate the particle diameter and zeta potential analyses, performed in triplicate and expressed in standard deviation.^25^

### Freeze–thaw Cycle

The optimized nanoemulsions were frozen at −20 °C for 24 h, then thawed at room temperature. The cycle was repeated three times and evaluated for macroscopic stability.^26^

### Heating Cooling Cycle

The prepared optimized formulation was characterized for the cooling–heating cycle to check the thermodynamic stability of the nanoemulsion. The nanoformulations were stored at 4 °C for 24 h and then kept for 24 h at 37 °C; the heating–cooling cycle was repeated three times.^27^

### Creaming and Cracking

The nanoemulsions were packaged and evaluated for creaming and cracking status after formulating. Thirty mL of each nanoemulsion was placed in a glass tube with a height of 65 mm and an internal diameter of 25 mm, left to rest for 24 h at 25 ± 2 °C, and then examined for physical characteristics. Cracking is characterized by physical instability and can be referred to as the permanent/irreversible partition or separation of the internal/dispersed phase (where the separation of water and oil is observed) on the surface of the nanoemulsion. If the nanoemulsions presented separations into cream and serum layers, the percentage of cream was determined by calculating the height of the cream layer (top layer) and the total height of the formulation using eq 1 given^28,29^
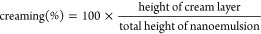
1

### Dilution Test and Transmittance Percentage

A dilution test was performed to observe the phase inversion of the nanoemulsions. Nanoemulsion was diluted 1:10 with deionized water in a test tube and observed for phase inversion. Furthermore, the percentage transmittance of the nanoemulsion was evaluated at 332 nm (%T), and deionized water was used as blank with the Ultrospec 3000 pro spectrophotometer (Amersham Pharmacia Biotech). The turbidity of the diluted nanoemulsions was calculated by taking the white control as 100% and using the following eq 2(30,31)
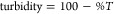
2

### Macroscopic Stability

The prepared formulation’s stability was determined using the centrifugation method.^32^ The nanoemulsions postformulated were subjected to centrifugation analysis to check the kinetic stability. Formulations were centrifuged at 1000, 2000, and 3000 rpm for 10 min. After that, the macroscopic stability was determined by comparing the aspects of formulation before and after the centrifugation cycle.

### Fourier Transform Infrared (FTIR) Spectroscopy

FTIR measurements were performed at 20 °C using the Jasco 4600 FTIR spectrophotometer with attenuated total reflectance (ATR) accessory. Each spectrum was acquired in the 400–4000 cm^–1^ spectral range at 0.4 cm^–1^ resolution. Ten μL of the nanoemulsion and balsam oil was added to the ATR support, and immediate readings were taken. Origin Pro 8.5 Software was used to visualize the spectra.^33^

### Electron Microscopy

The surface morphology of the developed nanoemulsions was analyzed using MORGAGNI 268 D transmission electron microscopy (FEI Company). A drop of the nanoemulsion was deposited on the grid coated with holey carbon film. After fixation, the grid with the sample was placed on a drop of 5% uranyl for 3 min, followed by three washes of the grid in ultrapure water.^34^

### Toxicity Assessment in *C. elegans*

#### *C. elegans* Maintenance

The wild-type *C. elegans* strain N2 (Bristol) and the *E. coli* feeding strain OP50 were obtained from the Caenorhabditis Genetics Center (CGC) (Minneapolis, MN, United States). Nematodes were maintained at 20 °C in nematode growth medium (NGM) agar plates seeded with live OP50 bacteria. For each experiment, synchronized populations were obtained through bleach treatments of gravid adults.^35^

#### Survival Assay

To assess the nanoemulsion toxicity on the survival of *C. elegans*, the animals were exposed to nanoemulsions of 0.1 and 2.5 mg mL^–1^, and counting of dead and alive worms after 24 h of exposure. The data obtained was then converted and expressed in percentage of survival. The tests were performed on ∼20 animals per group at a temperature of 20 ± 2 °C in duplicate, and four independent experiments^35,36^ For both survival and behavioral assays, OP50 bacteria were inactivated by 15 min exposure to UV–C light.^37^

### Behavioral Assay

#### Pharyngeal Pumping

A Pharyngeal pumping assay was performed to evaluate the feeding behavior of the worms when exposed to the nanoemulsions. The counting was made three times for each 10 worms for 10 s, and then the average number of pumps/10 s was calculated. Afterward, this number was normalized in pumps/min. The tests were performed at a temperature of 20 ± 2 °C using six animals per group and four independent experiments.^38,39^

#### Defecation Cycle

Defecation assays were performed at 22 ± 2 °C as previously described.^40^ Animals in the L4 larval stage on plates with *E. coli* were monitored, and the interval between defecations was identified. The average of three defecation cycles for each animal was used to indicate intestinal activity. Six worms per group were used in duplicate, and experiments were repeated at least four times.

#### Egg Production

Egg production was evaluated in gravid animals (adult day 1, ∼72 h after the treatment). The number of eggs in the worms’ uterus was determined by individual counting after lysing the animals with the bleaching solution. The tests were performed on 8 animals per group at a temperature of 20 ± 2 °C in duplicate, and four independent experiments.^40^

#### Swimming

After chronic treatment, nematodes were washed three times with an M9 buffer to remove bacteria. Afterward, the animals were placed on 96-well plates with M9 at 20 °C, and after acclimatization, body movements were counted. The swimming assay was evaluated in ten worms at L4 larval and four independent experiments, and the number of body bends was counted during the 20 s and normalized per minute.^41,42^

#### Body Length

A body length assay was used to evaluate the worms’ development after exposure to the nanoemulsions at 0.1 and 2.5 mg mL^–1^. The body measures were acquired from ten L4 animals per group at a temperature of 20 ± 2 °C in duplicate and four independent experiments. ImageJ software (National Institutes of Health—NIH) allowed the obtained picture analysis.^43^

### Statistical Analysis

All statistics and graphs were performed in GraphPad Prism version 10.2. The assessment of the significance of statistical analysis was performed by One-Way and Two-way Analysis of Variance (ANOVA), followed by posthoc Bonferroni’s test. To set a significance level, a *p* < 0.05 was determined to represent the statistical difference between means.

## Results and Discussion

### Chromatography of Copaiba Balsam Oil and Nanoemulsion

GC/MS of Copaiba balsam oil identified the occurrence of compounds such as Caryophyllene, the majority constituent in Copaiba balsam oil, being the most abundant group with 87.44%. Copaene was the second most abundant compound with 2.98%, followed by α-Humullene (2.58%), β-Bisabolene (1.37%), β-Costol (1.05%), and δ-Cadinene (1.02%), respectively (Table 1). The technique presented resolution and separation of peaks, allowing the compounds to be analyzed individually in databases of spectra libraries and retention times found in the literature (Figure S1, Supporting Information).

**Table 1:** Chemical Composition of Copaiba Balsam Oil Obtained by GC/MS

number	name	RT (min)	area (%)	CAS number
1	δ-eIemene	20.711	0.12	20 307-84-0
2	α-cubenene	21.186	0.43	17 699-14-8
3	copaene	22.322	2.98	3856-25-5
4	β-elemene	22.537	0.38	515-13-9
5	isocaryophyllene	23.437	0.24	118-65-0
6	caryophyllene	24.028	87.44	87-44-5
7	10,10-dimethyl-2,6- dimethylenebicyclo[7.2.0]undecane	24.218	0.37	357 414-37-0
8	α-humullene	25.168	2.58	6753-98-6
9	γ-muurolene	25.568	0.51	30 021-74-0
10	β-(*Z*)-farnesene	25.759	0.13	28 973-97-9
11	β-copaene	25.979	0.39	18 252-44-3
12	β-bisabolene	26.234	1.38	495-61-4
13	selinene	26.569	0.24	473-13-2
14	α-elemene	26.954	0.24	5951-67-7
15	δ-cadinene	27.059	1.02	483-76-1
16	thujopsene	29.295	0.10	470-40-6
17	14-hydroxycaryophyllene	29.525	0.17	50 277-33-3
18	β-costol	29.680	1.05	515-20-8
19	junenol	31.106	0.11	472-07-1
20	T-cadinol	31.411	0.12	5937-11-1
	Total		100	

Other researchers also identified caryophyllene as the constituent with the highest concentration in samples of Copaiba balsam oil, corroborating our findings,^44^ as well as other molecules common to the oil.^45^

Likewise, the nanoemulsions’ chemical composition was evaluated to detect the percentage concentration of oil constituents in the formulations and observe possible changes in the profile of the molecules after the nanoformulation process. GC/MS of nanoemulsion of Copaiba balsam oil identified the occurrence of compounds such as Caryophyllene, the majority constituent that continued in higher concentration (32.5%) (Table 2, Figure S2, Supporting Information). This percentage decrease may be related to the reduction of the concentration of Copaiba balsam oil in the formulation, which corroborates the decline in the concentration of the majority of constituents.

**Table 2:** Chemical Composition of Nanoemulsion of Copaiba Balsam Oil by GC/MS

number	name	RT (min)	area (%)	CAS number
1	pentane, 3-methyl-	4.259	1.06	96-14-0
2	(3-methyl-oxiran-2-yl)-methanol	4.429	0.99	
3	2,2-dimethoxybutane	7.075	1.42	3453-99-4
4	toluene	7.355	1.22	108-88-3
5	cyclotrisiloxane, hexamethyl-	8.360	0.90	541-05-9
6	stannane, bicyclo[4.2.0]octa-1,3,5-trien-7-yltrimethyl-	8.415		
7	*p*-xylene	9.561		106-42-3
8	undecane, 2,6-dimethyl-	13.993	0.90	17 301-23-4
9	γ-cubebene	22.307	0.90	17 699-14-8
10	caryophyllene	24.013	32.56	87-44-5
11	humulene	25.153		6753-98-6
12	trisiloxane, 1,1,1,5,5,5-hexamethyl-3,3-bis[(trimethylsilyl)o xy]-	29.790	0.63	3555-47-3
13	9-octadecenoic acid	33.312	1.18	2027-47-6
14	2,6-dihydroxyacetophen one, 2TMS derivative	37.869	1.12	
15	2,3,5,6-tetrafluoro-4-methoxybenzoic acid, TMS	38.494		
16	4-tert-octylphenol, TMS derivative	39.580	1.45	78 721-87-6
17	2,6-dihydroxyacetophen one, 2TMS derivative	39.645	1.48	
18	trans-4,4′-dimethoxy-betamethylchalcone	40.060		61 000-04-2
19	1,2-benzisothiazol-3-amine, TBDMS derivative	40.650	1.79	
20	trans-4-(2-(5-nitro-2-furyl)vinyl)-2-quinolinamine	41.045	2.22	847-10-9
	total		49.8	

The conservation of the following constituents was observed in the GC-MS analysis of the Copaiba balsam oil nanoemulsion.

### Average Particle Size

**Figure 1:**
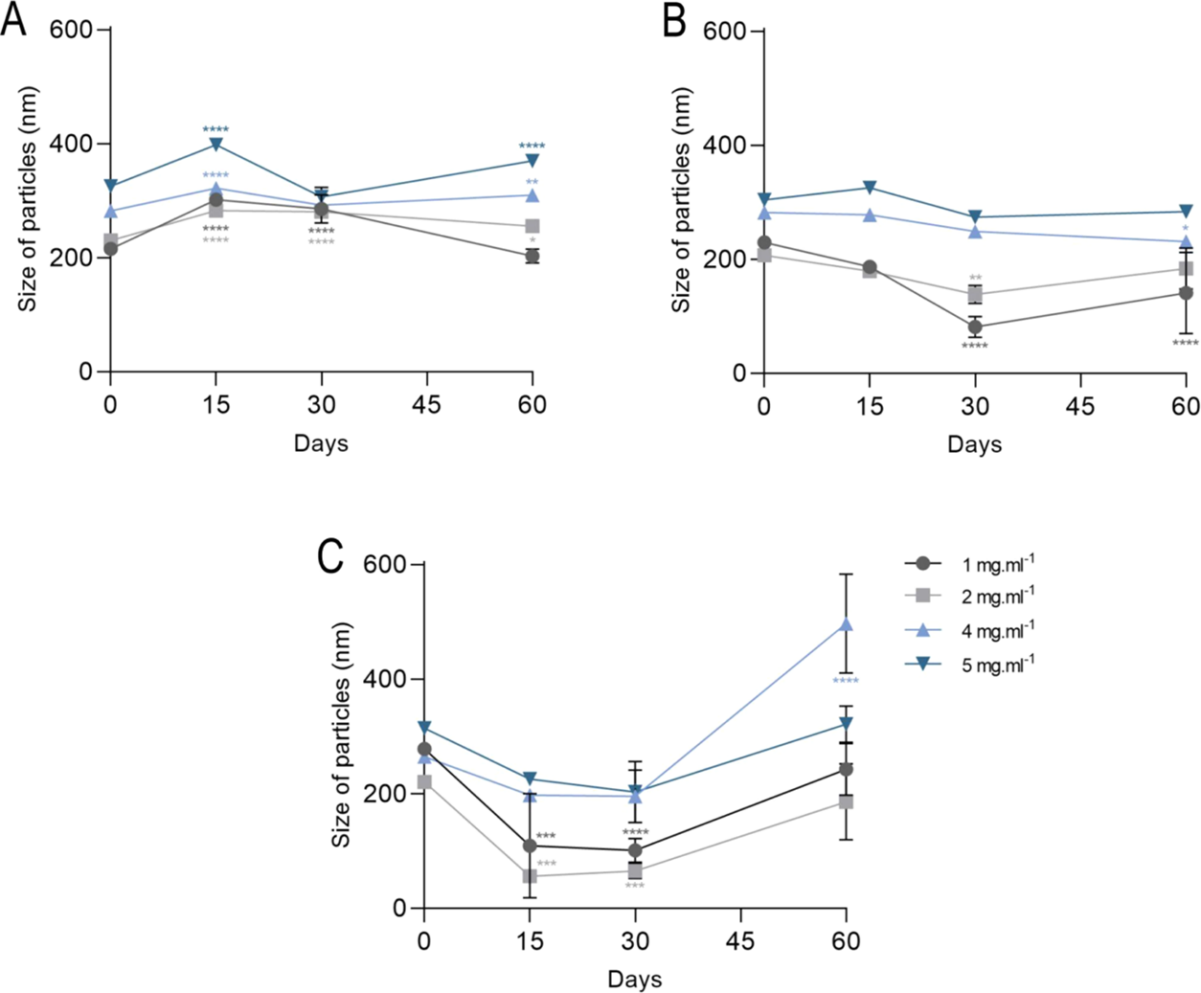
Characterization of particle size of Copaiba balsam oil nanoemulsions of 1, 2, 4, and 5 mg mL^–1^ with final concentration of 0.05% tween 80 and stored for 60 days at (A) 4 ± 2, (B) 20 ± 2, and (C) 36 ± 2 °C. Data are represented as mean ± SD. Statistical significance was determined by Two-way ANOVA followed by Bonferroni’s test. *p < 0.05, **p < 0.01, ***p < 0.001, and ****p < 0.0001.

The prepared nanoformulations under three different temperature conditions (4 ± 2, 20 ± 2, and 36 ± 2 °C) showed stability immediately after formulation since there were no creaming, phase separation, and/or changes in macroscopic findings related to the destabilization of the system (Figure 1). All nanoemulsions stored at 4 ± 2 °C showed an average particle size between 215.8 and 398.5 nm, presenting different particle sizes at the end of the 60 days of evaluation. Nanoemulsions at 1, 2, 4, and 5 mg mL^–1^ showed final particle sizes after 60 days of 203.3, 256.1, 310.3, and 370.3 nm, respectively. At a temperature of 20 ± 2 °C, mean sizes from 81.9 to 325.9 nm were observed over time, where during the 60 days (Figure 1B). Formulations stored at a higher temperature, such as 36 ± 2 °C, showed greater instability over time (Figure 1C).

In general, the nanoemulsions stored at 4 ± 2 °C showed less variation in droplet diameters (Figure 1A). In nanoemulsions at 4 ± 2 °C, the 1 mg mL^–1^ formulation droplet size was statistically significant when compared to 15 (*p* < 0.0001) and 30 days (*p* < 0.0001). At 2 mg mL^–1^, there was an increase in particles after 15 (283.2 nm, *p* < 0.0001), 30 (281 nm, *p* < 0.0001), and 60 days (256.1 nm, *p =* 0.01). The 4 mg mL^–1^ formulations showed slight increases after 15 (322.2 nm, *p* < 0.0001) and 60 days (310.3 nm, *p =* 0.0069). The 5 mg mL^–1^ nanoemulsion achieved a significant increase after 15 days (398.5 nm, *p* < 0.0001), returning to the initial particle size on day 30 and increasing again after 60 days (370.3 nm, *p* < 0.0001).

Nanoemulsions stored at 20 ± 2 °C showed variations over time. The 1 mg mL^–1^ formulation, decreased in size after 30 (81.9 nm, *p* < 0.0001) and 60 days (141.4 nm, *p* < 0.0001). Similarly, the 2 mg mL^–1^ nanoformulation also showed a decrease in particle size. The 4 mg mL^–1^ nanoemulsion showed a decrease in particle size only after 60 days (231.7 nm, *p* < 0.03), and the 5 mg mL^–1^ formulation showed no significant changes. At 36 ± 2 °C, the 1 mg mL^–1^ formulations, showed a reduction after 15 (109.3 nm, *p* < 0.0002) and 30 days (101.6 nm, *p* < 0.0001). A similar reduction was observed in 2 and 5 mg mL^–1^. At 4 mg mL^–1^, only after 60 days was there a significant increase in size (497.3 nm, *p* < 0.0001).

The thermodynamic instability of nanoemulsions may enable destabilization under unfavorable environmental conditions.^46^ The size of the droplets at high temperatures may be due to the Brownian motion resulting in flocculation and/or coalescence, given that the centrifugal force and high temperatures accelerate the Brownian motion and increase particle size.^47^ Researchers also observed the destabilization of nanoemulsions by increasing temperature.^48^ Also was detected that Copaiba balsam oil nanoemulsions stored at 4 °C showed no change in macroscopic characteristics, which may indicate the stabilization of the particles.^49^ In general, our nanoemulsions stored at 4 and 20 ± 2 °C showed the most minor variations in particle size.

The process of agitation and shear forces provides the energy needed for the initial emulsion system, where intense agitation reduces the size of the oil droplets.^50^ Oily nanoparticles are reduced due to deforming forces generated by high energy that overcomes Laplace pressure and decrease their size.^51^ To the best of our knowledge, our Copaiba balsam oil nanoemulsions are the only ones with low surfactant levels in the literature. Tween 80 has been associated with adverse events, such as changes in the translocation of intestinal bacteria, and systemic allergic reactions, such as skin rash and hypersensitivity, which makes it necessary to reduce the use of surfactant.^52^

Storage time and temperature of nanoemulsions can affect viscosity through crystallization of the oil phase, inducing partial coalescence of dispersed droplets or contributing to conformational modifications of surfactant molecules.^53,54^ These events may have contributed to the changes in particle size over time observed in our study.^53^ Increasing the temperature in a nanosystem increases the kinetic energy of the molecules, leading to greater droplet movement and a greater tendency to coalescence.^55^ High temperatures tend to reduce the viscosity of the nanoemulsion, also contributing to changes in physical-chemical characteristics.^56^ Furthermore, the mass transport kinetics of oil and surfactant molecules are altered with viscosity modifications influenced by different temperatures; this phenomenon may also be related to the variations in particle size observed in our study.^53^

### Polydispersity Index (PdI)

**Figure 2:**
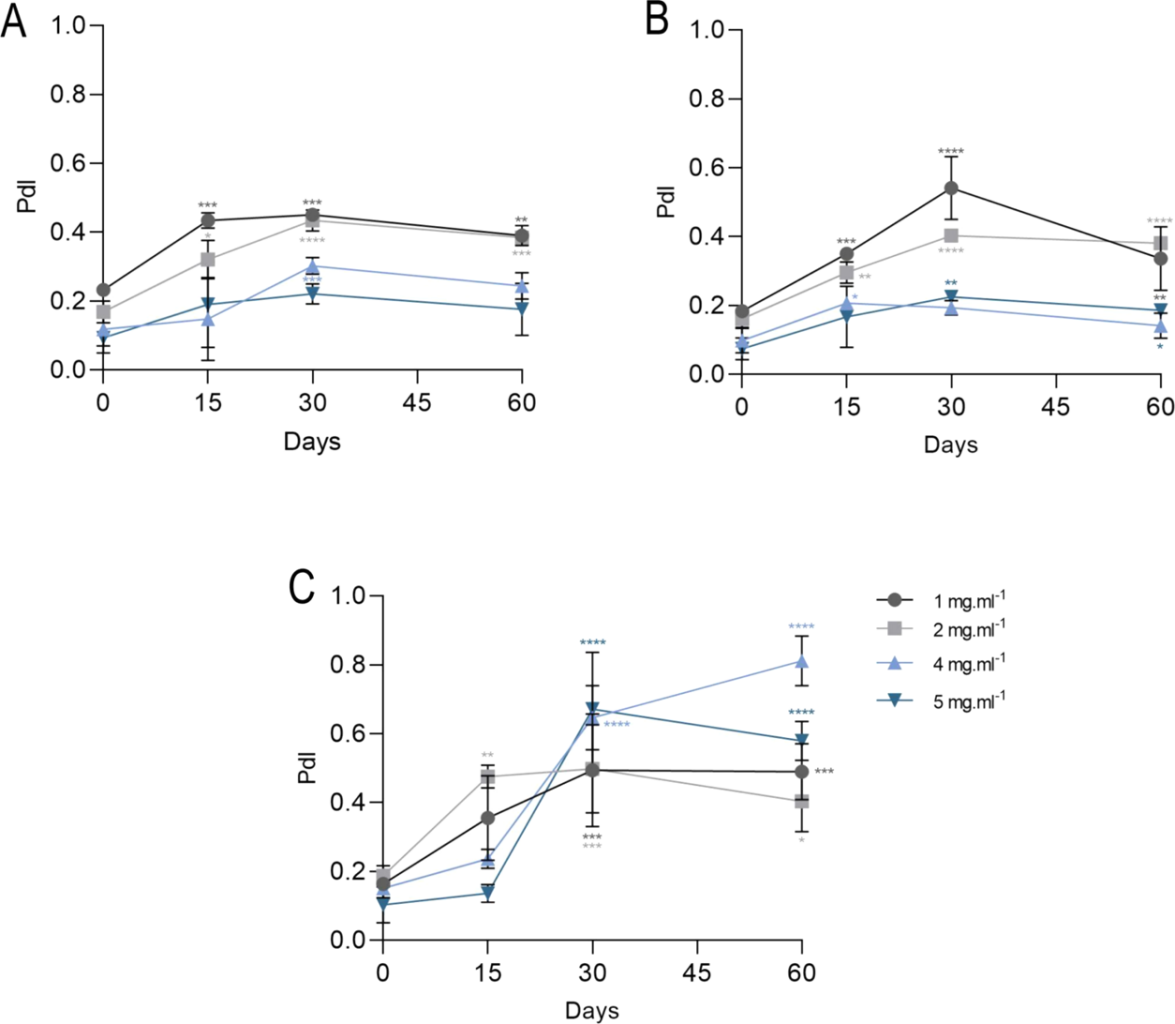
Characterization of polydispersity index of Copaiba balsam oil nanoemulsions of 1, 2, 4, and 5 mg mL^–1^ with final concentration of 0.05% tween 80 and stored for 60 days at (A) 4 ± 2, (B) 20 ± 2, and (C) 36 ± 2 °C. Data are represented as mean ± SD. Statistical significance was determined by Two-way ANOVA followed by Bonferroni’s test. *p < 0.05, **p < 0.01, ***p < 0.001, and ****p < 0.0001.

Nanoformulations PdI presented values between 0.1 and 0.4 during all evaluation periods (Figure 2). Nanoemulsions stored at 4 ± 2 °C showed PdI values between 0.09 after formulation and 0.3 at the end of the 90 days. The 1 mg mL^–1^ nanoemulsion showed significant differences in terms of PdI, as there were increases after 15 (0.4, *p =* 0.0006), 30 (0.4, *p =* 0.0002) and 60 days (0.3, *p =* 0.009) of storage. A similar increase was observed in 2 mg mL^–1^ nanoemulsions. At 4 mg mL^–1^, a significant increase was observed only after 30 (0.3, *p =* 0.001), and the 5 mg mL^–1^ nanoemulsions did not show significant differences over time (Figure 2A).

Nanoemulsions stored at 20 ± 2 °C showed PdI between 0.07 and 0.5, still final polydispersions in 90 days with values ranging from 0.1 to 0.3. The 1 mg mL^–1^ nanoformulation showed an increase in PdI 15 (0.3, *p =* 0.0004), 30 (0.5, *p* < 0.0001), and 60 days (0.3, *p =* 0.0012) after formulation. At 2 mg mL^–1^, there was also an increase after 15 (0.2, *p =* 0.0048), 30 (0.4, *p* < 0.001), and 60 (0.3, *p* < 0.0001) days. The 4 mg mL^–1^ nanoformulations also achieved increases in PdI just 15 days (0.2, *p =* 0.032) after formulation and changes 30 (0.2, *p =* 0.0014) and 60 days (0.1, *p =* 0.0254) after formulation in the nanoemulsion at 5 mg mL^–1^ (Figure 2B). The nanoemulsions at 35 ± 2 °C had the greatest variations, presenting final values of 0.4–0.8 PdI.

The polydispersity is related to the degree of uniformity of a particle size distribution.^57^ The dynamic light scattering technique requires that nanoformulations have polydispersity values below 0.1 and are considered highly monodisperse, and values from 0.1 to 0.4 are considered moderately dispersed.^58^ Even though our nanoemulsions showed changes in PdI over storage time, they remained within the recommended range for uniform nanoemulsions. Changes in PdI during storage periods may be due to oil and surfactant concentrations. When the oil concentration is changed by fixed volumes of surfactant, fewer surfactant molecules are available to coat the oil particle, and due to increased interfacial tension, coalescence and increased PdI can occur.^26^ However, even with significant changes in PdI in formulations with low surfactant concentrations, the nanoemulsions remained within the desirable quality range.

### Zeta Potential

**Figure 3:**
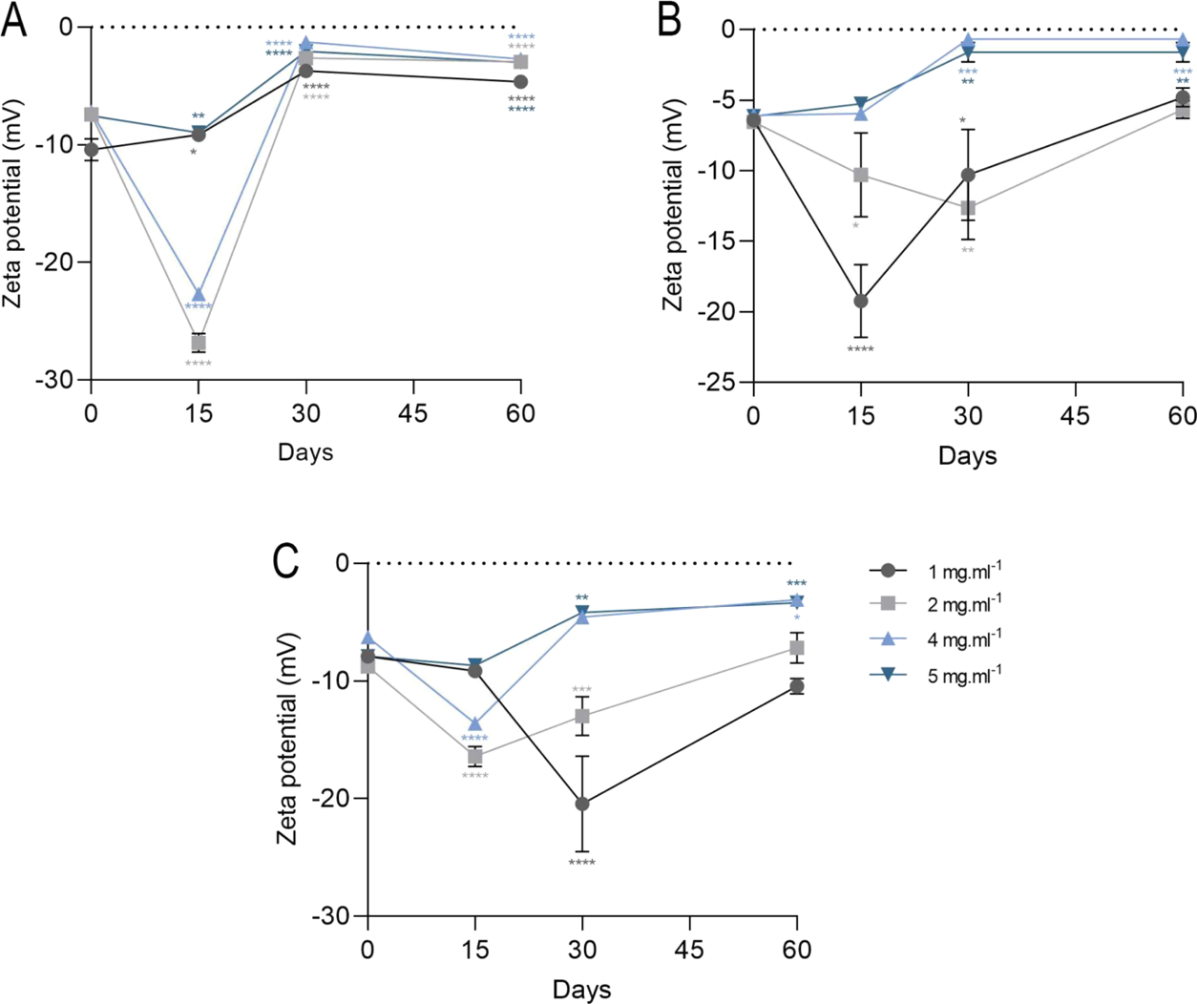
Characterization of zeta potential of Copaiba balsam oil nanoemulsions of 1, 2, 4, and 5 mg mL^–1^ with final concentration of 0.05% tween 80 and stored for 60 days at (A) 4 ± 2, (B) 20 ± 2, and (C) 36 ± 2 °C. Data are represented as mean ± SD. Statistical significance was determined by Two-way ANOVA followed by Bonferroni’s test. *p < 0.05, **p < 0.01, ***p < 0.001, and ****p < 0.0001.

The nanoparticles were diluted in saline solution with pH 6 and showed negative zeta potential values between −6 and −8 mV in the immediate postformulation period (Figure 3). However, formulations stored at 4 ± 2 °C showed a decrease in zeta potential in the period of the 15 days of storage, where the formulations of 4 and 2 mg mL^–1^ obtained values of −22 (*p* < 0.0001) and −26 mV (*p* < 0.0001) respectively, increasing on 60 days (Figure 3A). Nanoformulations stored at 20 ± 2 °C showed variations over the days of storage, with a concentration of 1 mg mL^–1^ showing a marked decrease in the zeta potential in the 30 days (−20.4 mV, *p* < 0.0001), returning to −10 mV (*p =* 0.0012) within 60 days (Figure 3B). Storage at 36 ± 2 °C varied the surface electrical potential of the nanoemulsion, where the 1 mg mL^–1^ formulation had an average value of −19 mV (*p* < 0.0001) on the 30 days of analysis, with all nanoemulsions between −6 and −5 mV at the end of the 60 days of analysis (Figure 3C).

The observed variation in zeta potential, such as decreases in surface charge, can be attributed to variations due to chemical changes, such as decomposition of surfactant or formation of charged molecules.^53^ Furthermore, the increase in negativity observed on some days of the nanoemulsion analysis may also be related to differences in dissociation and the number of ionizable compounds in copaiba balsam oil in the formulation.^53^

The zeta potential plays a role in the integration of particles into the cell membrane and is important for the stability of nanoparticles in suspension.^59^ The surface charge of nanostructures plays a key role in cell adsorption, where the adsorption process follows two major moments: the binding of the nanoparticle to the cell membrane and cell internalization.^60^ Nanoparticles with zeta potential between −10 and +10 mV are considered approximately neutral, and nanoparticles with a surface charge above +30 mV and below −30 mV are considered strongly cationic and anionic, respectively.^61^ The zeta potential directly affects the permeation of nanoparticles in the cell membrane, considering that cell membranes are negatively charged and cationic particles exhibit greater toxicity causing the cell membrane to rupture.^61^

The surface charge of particles is also necessary for the interaction with cell membranes and biological macromolecules. Nanoparticles can undergo protein surface adsorption mechanisms, leading to agglomeration and elimination of particles by the reticuloendothelial system and making reaching the target region difficult. Some studies have shown that nanoparticles with a neutral surface charge can provide a valuable route to minimize undesirable interactions of nanoparticles with the biological environment.^62^

### Stability of Nanoemulsions after One and Two Years of Analysis

**Figure 4:**
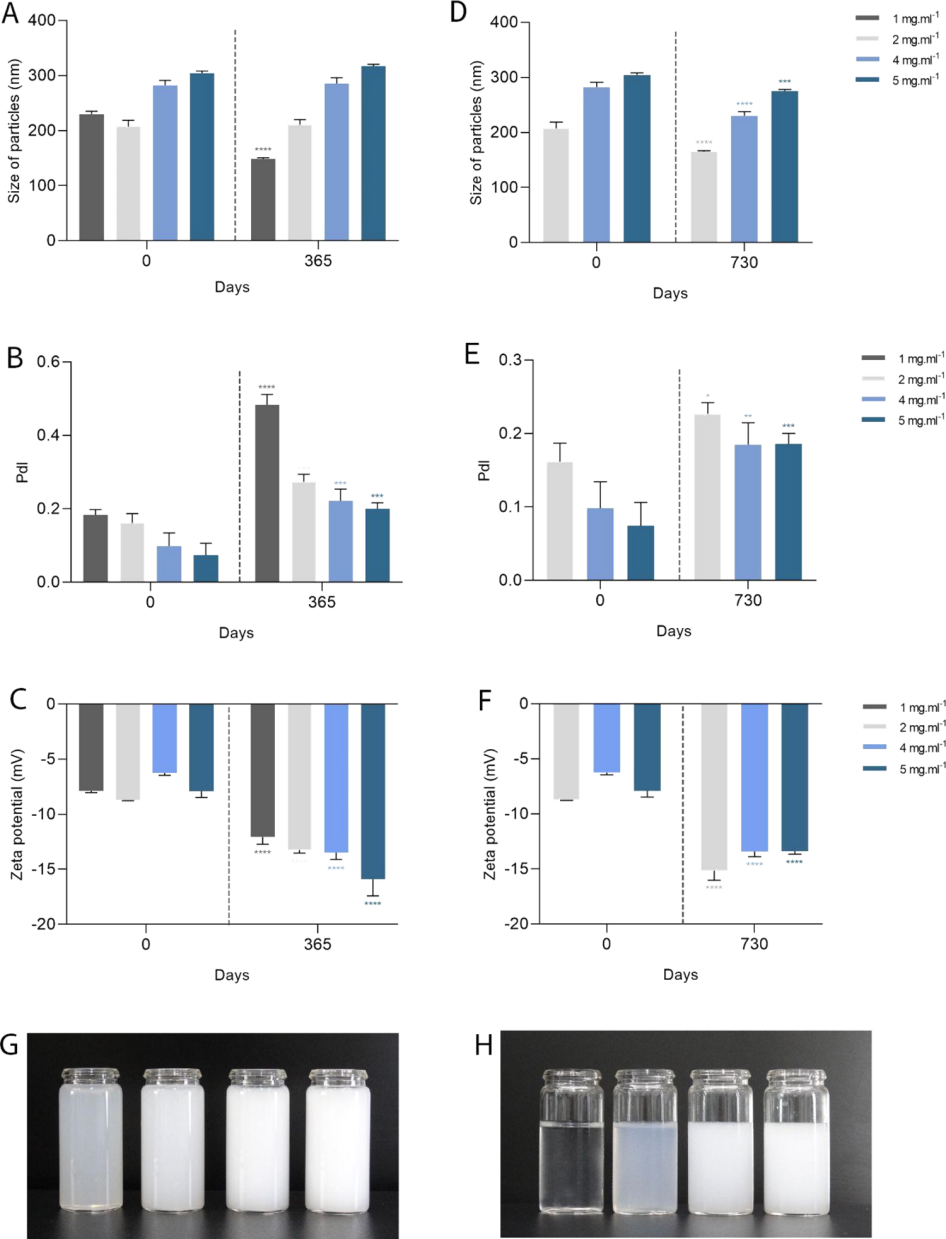
Characterization of particle size, polydispersity index, and zeta potential of Copaiba balsam oil nanoemulsions of 1, 2, 4, and 5 mg mL^–1^ with a final concentration of 0.05% tween 80 and stored for one (A–C) and two years (D–F) at 4 ± 2 °C, respectively. Nanoemulsions image immediately after formulation (G). Nanoemulsions image after two years (H). Data are represented as mean ± SD. Statistical significance was determined by Two-way ANOVA followed by Bonferroni’s test. *p < 0.05, **p < 0.01, ***p < 0.001, and ****p < 0.0001.

After one year of storage at 4 ± 2 °C, the nanoemulsions did not show significant differences since the particle sizes remained similar over time, except for the concentration of 1 mg mL^–1^ with a reduction of 229.9–148.7 nm (*p* < 0.0001) (Figure 4A). After two years (730 days) under storage at 4 ± 2 °C, the nanoemulsions showed a significant decrease in particle size, where the nanoformulations of 2 and 4 mg mL^–1^, showed a reduction of 207.3–165.2 nm (*p =* 0.005), and 282.3–230.3 nm (*p =* 0.0008), respectively. The 5 mg mL^–1^ nanoemulsion after 2 years did not show a significant change in particle size. The 1 mg mL^–1^ nanoemulsion did not meet the quality criteria for analysis on the equipment (Figure 4D,H).

In PdI, the nanoemulsions analyzed after one year showed significant increases in PdI. The nanoemulsion of 1, 2, 4, and 5 mg mL^–1^ presented after 1 year PdI of 0.4 (*p* < 0.0001), 0.2 (*p =* 0.0004), 0.2 (*p =* 0.0001), and 0.1 (*p =* 0.0001), respectively. However, they remained within the appropriate range for nanoformulations (Figure 4B). The same was observed in the nanoemulsions after 2 years of storage, with nanoemulsions of 2, 4, and 5 mg mL^–1^ obtaining final values of 0.2 (*p =* 0.0466), 0.1 (*p =* 0.0067), and 0.1 (*p =* 0.0007) (Figure 4E).

The zeta potential, however, became more negative after one year of storage. The concentrations of 1, 2, 4, and 5 mg mL^–1^ showed significant decreases, reaching −12 mV (*p* < 0.0001), −13.2 mV (*p* < 0.0001), −13.5 mV (*p* < 0.0001), and −15.9 mV (*p* < 0.0001), respectively, after 1 year (Figure 4C). After 2 years of storage, the nanoemulsions also showed a reduction in zeta potential. Concentrations of 2, 4, and 5 mg mL^–1^ achieved a significant reduction, reaching −10.3 mV, −15.1 mV (*p* < 0.0001), −13.4 mV (*p* < 0.0001), and −13.4 mV (*p* < 0.0001), respectively (Figure 4F).

After one year of storage, the nanoemulsions remained stable. However, the particle size in the nanoemulsion decreased significantly after two years. Other researchers also observed a decrease in particles over storage time after two years.^63^ The reduction in the size of our nanoemulsions can be attributed to the decrease in the number and size of particles in the system, suggesting that the Copaiba oil molecules were leaving the oil droplets and entering the surfactant micelles.

The PdI of the nanoformulations after one and two years showed significant increases. As mentioned previously, when particles coalesce, an increase in PdI is observed, and this increase may occur due to destabilization processes. However, although changes were observed, they remained within the expected standards for stable nanoformulations. Ostwald ripening is one mechanism that can occur in nanoemulsions stored for long periods of time, which is based on the incorporation of smaller particles into larger particles. In our nanoemulsions with changes in PdI, we hypothesize that Brownian motion and random collisions between particles can cause the inclusion of smaller particles into larger ones.^64^

### Stability of Nanoemulsions after Blood, Gastric, and Intestinal pH

**Figure 5:**
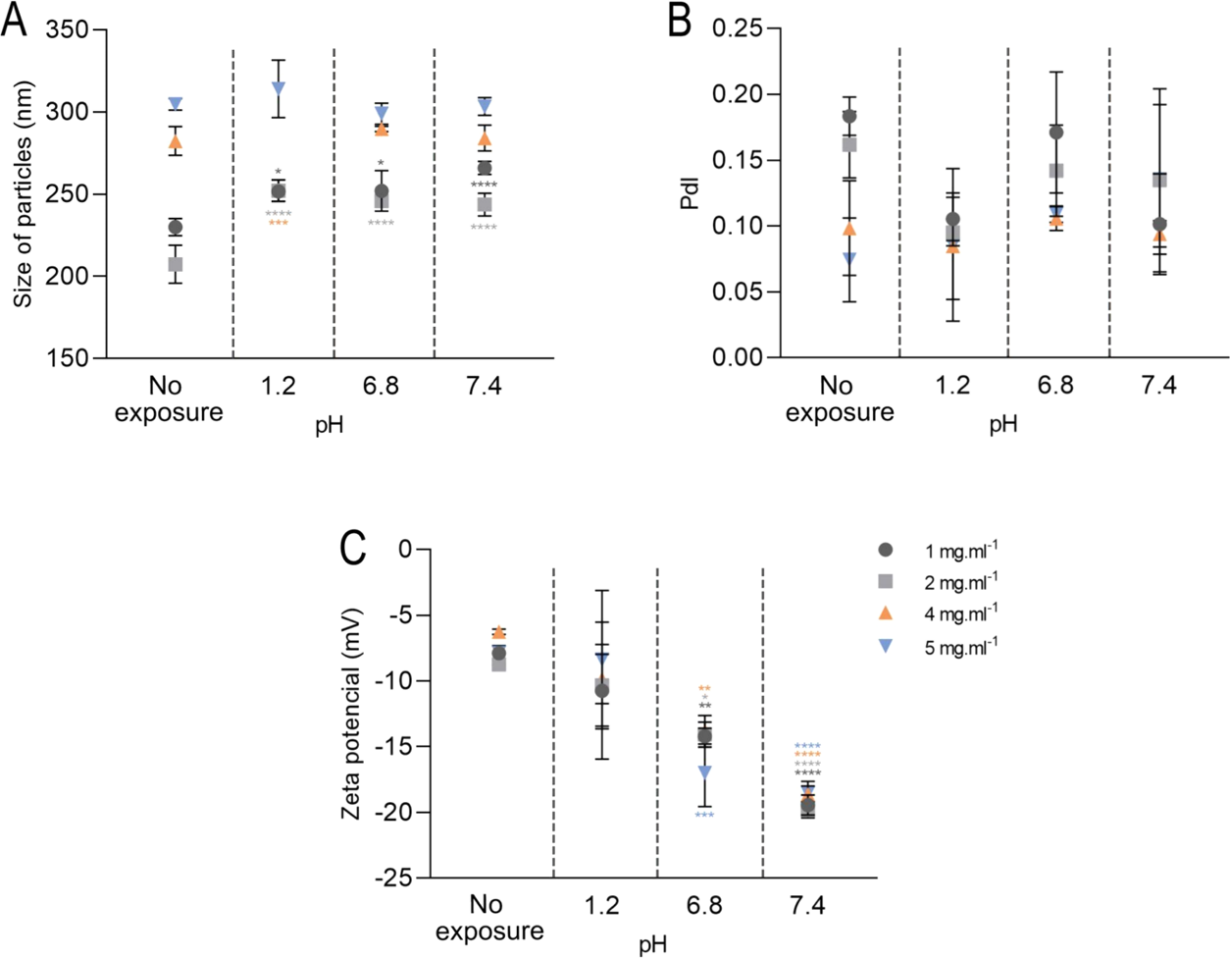
Characterization of nanoemulsions of Copaiba balsam oil not exposed to different pH and exposed to pH 1.2, 6.8, and 7.4 by particle size (A), polydispersity index (B), and zeta potential (C) at 1, 2, 4, and 5 mg mL^–1^ with final concentration of 0.05% tween 80 after formulation. Data are represented as mean ± SD. Statistical significance was determined by Two-way ANOVA followed by Bonferroni’s test. *p < 0.05, **p < 0.01, ***p < 0.001, and ****p < 0.0001.

The pH of the nanoemulsions after formulation was around 5 in normal preparation conditions (Table S1, Supporting Information). Stability analysis at different pH after exposure of the nanoemulsions to gastric pH (pH 1.2); in particle size, we observed changes in the nanoformulations at concentrations of 1, 2, and 4 mg mL^–1^, with sizes from 229.9 to 252.2 nm (*p =* 0.0125), 207.3 to 252.2 nm (*p* < 0.0001) and 282.3 to 252.2 nm (*p =* 0.0003), respectively. The 5 mg mL^–1^ nanoemulsion showed no significant difference (Figure 5A).

At intestinal pH (pH 6.8), the 1 and 2 mg mL^–1^ nanoemulsions showed significant increases in size, from 229.9 to 252 nm (*p =* 0.0107) and 207.3 to 245.8 nm (*p* < 0.0001), respectively. The size of the nanoparticles at concentrations of 4 and 5 mg mL^–1^ did not show significant differences compared to the baseline value (Figure 5A). In blood pH (pH 7.4), there was also a significant difference in the 1 and 2 mg mL^–1^ nanoemulsions, showing increases from 229.9 to 266 nm (*p* < 0.0001) and 207.3 nm to 243.7 (*p* < 0.0001), respectively. The concentrations of 4 and 5 mg mL^–1^ showed no significant differences (Figure 5A).

The PdI of the nanoemulsions remained low and did not show significant differences (Figure 5B). However, the surface charge of the nanoparticles showed important reductions. In gastric pH, the nanoemulsions did not show significant differences compared to baseline values. In intestinal pH, nanoemulsions of 1, 2, 4, and 5 mg mL^–1^ obtained values of −14.2 mV (*p =* 0.0093), −14 mV (*p =* 0.0387), −13.8 mV (*p =* 0.0014) and −17 mV (*p =* 0.0001) with significant differences, respectively. At blood pH, the nanoformulations significantly reduced the surface charge, becoming more negative where concentrations of 1, 2, 4, and 5 mg mL^–1^ presented values of −13.2 mV (*p* < 0.0001), −14 mV (*p* < 0.0001), 13.8 mV (*p* < 0.0001) and 18.5 mV (*p* < 0.0001) compared to baseline values, respectively (Figure 5C).

pH modifications, such as acidic pH, can modify the surface charges of the molecules that make up vegetable oil. Furthermore, surfactants can undergo ionization or conformational changes that can interfere with particle stability and alter surface adsorption.^65^

The increase in particle size observed at concentrations of 1 and 2 mg mL^–1^ may have occurred due to aggregation mechanisms, although there was a significant decrease in surface charge. At pH 6, tween 80 was observed to increase the colloidal stability of branched-chain amino acid nanosuspensions dramatically.^66^ Furthermore, tween 80 hydrolysis rates are increased at pH below 3 and above pH 7.6.^67^ The decrease in surface charge may be due to the increase in pH, which corroborates the reduction in free H^+^ levels, resulting in lower H^+^ concentrations and, consequently, greater surface charge on the particle surface.^68^

### Viscosity

In general, the freshly formulated nanoemulsions presented a viscosity close to that of water at a temperature of 25 °C. The nanoemulsion has presented viscosity of 1.14 mPas.s after formulations since the nanoemulsions of 1, 2, 4, and 5 mg mL^–1^ presented 1.14 ± 0.05, 1.14 ± 0.05, 1.14 ± 0.04, and 1.14 mPas.s (Table S1, Supporting Information). Our nanoemulsions have low viscosity considering that the dispersed phase is composed mostly of water and a small concentration of oil is added.

The relative viscosity of two phases, dispersed (oil) and continuous (water), strongly influences particle size reduction. When the nanoformulation’s viscosity is very high, the oily particles become resistant to rupture, rotating around their axis and decreasing mobility in the continuous phase.^69^ Increasing water content during formulation reduces viscosity.

### Fourier Transform Infrared (FTIR) Spectroscopy

The mixture of oil, bioactive compounds, surfactants, physical bonds, and chemical interactions are reflected by changes in characteristic bonds observable in FTIR.^70^ IR spectroscopy is based on determining the energy difference (Δ*E*) between the excited and ground states of molecules.^71^

**Figure 6:**
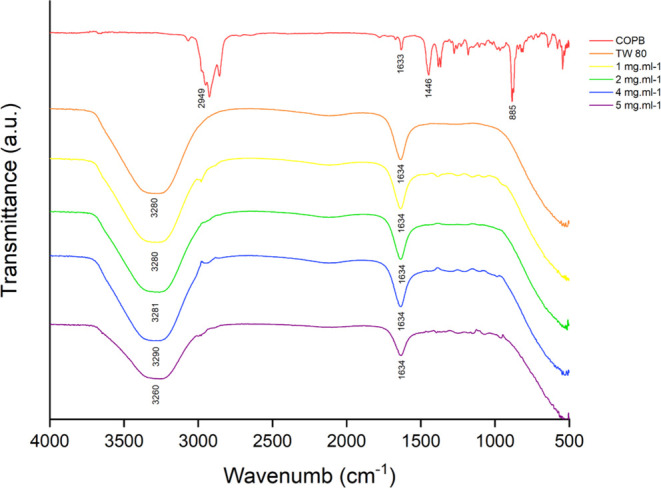
FTIR spectrum of Copaiba balsam oil nanoemulsions of 1, 2, 4, and 5 mg mL^–1^ and tween 80.

FT-IR analyses were carried out to evaluate the interaction between copaiba oil and the other constituents of the formulation (Figure 6). The IR spectrum of copaiba oil showed in the region of 2949 cm^–1^ bands characteristic of symmetric and asymmetric CH_2_ bonds. In the region close to 1633 cm^–1^, an angular deformation C=C was observed, characteristic of monoterpenes, and in the region of 1446 cm^–1^, bands characteristic of sesquiterpenes. In the region close to 885 cm^–1^, characteristic bands of monoterpenes with out-of-plane angular deformation. Furthermore, the IR of Tween 80 showed characteristic bands at a wavelength of 3280 cm^–1^, referring to the OH groups. Furthermore, it also presented characteristic bands close to the 1634 cm^–1^ region, referring to the presence of alkene groups, of the C=C type.

After the interaction of copaiba oil with Tween 80, it is possible to notice an overlap of the band referring to the hydroxyl groups (3450 cm^–1^) of Tween 80 over the band referring to the CH_2_ bonds (2948 cm^–1^) of copaiba oil, so that this overlap is due to intermolecular interactions between these two constituents. Intermolecular interactions can also be evidenced in the overlap of the 1634 cm^–1^ band referring to the alkene group of Tween 80 over the 1633 cm^–1^ band referring to the monoterpenes of Copaiba balsam oil. It is worth highlighting that in the presence of Tween 80, the peaks 1446 and 885 cm^–1^ referring to mono and sesquiterpenes, respectively, show a reduction in emission intensity. These data reveal high homogeneity of the nanoemulsion, even with an increase in the concentration of Copaiba balsam oil.

Other researchers also identified spectra similar to Copaiba balsam oil used in nanoemulsions. In another copaiba oil, bands were identified in the regions between 2924 cm^–1^ (−CH alkanes) and 2852 (−CH aldehydes) close to the bands identified in our oil. Furthermore, were identified bands between 1693 and 887 cm^–1^, nearby vibrational bands, and in the spectra of the formulations were also identified bands in the region 3300 and 1640 cm^–1^, where our nanoemulsions presented similar vibrational bands.^72^ Also, the vibrational bands of 2951 cm^–1^ identified in another study were close to those identified in our balsam oil.^73^

### Electron Microscopy and Size Distribution

**Figure 7:**
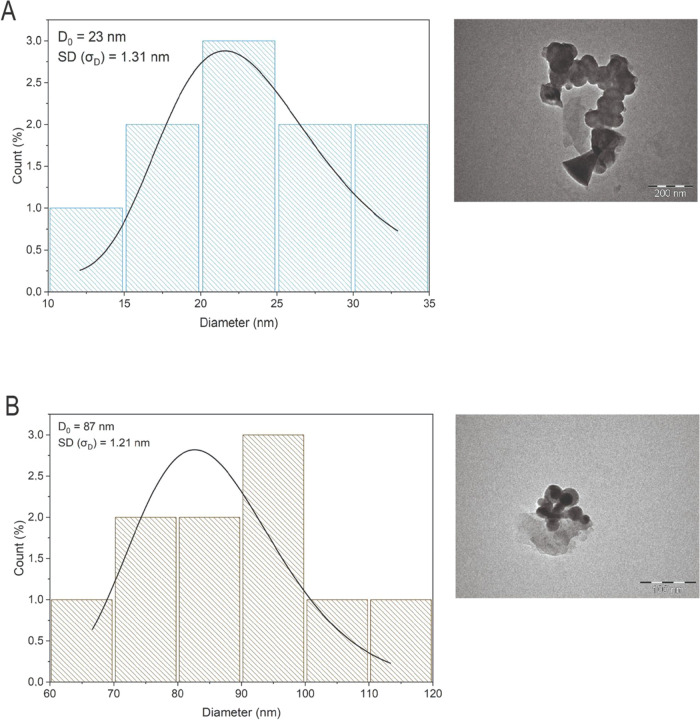
Transmission electron microscope micrograph and particle size distribution of 2 (A) and 5 mg mL^–1^ (B) nanoemulsions. Particle size distribution of nanoformulations annealed with a log-normal distribution function.

The nanoparticles that make up the nanoemulsion have a spherical shape; although agglomerated, it is possible to observe this distinction (Figures 7 and S3). The same morphological findings were observed in another nanoemulsion of Copaiba oil^74^ and different vegetable oils.^75−77^

The particles were not homogeneous. However, they could be analyzed individually in terms of shape and diameter. The particle sizes of 2 and 5 mg mL^–1^ nanoemulsions were estimated by fitting the distribution histogram to the log-normal distribution function, represented by the following eq 3
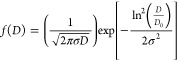
3where *D* corresponds to the average particle size and σ_D_ is the standard derivation.^78^

The nanoemulsions presented average size values of 23 and 87 nm at 2 and 5 mg mL^–1^ concentrations, respectively (Figure 7). The difference in the average particle size in dynamic light scattering (DLS) and transmission electron microscopy (TEM) may be due to particle aggregation during DLS analyses.^79^ In DLS, surfactant and water molecules surrounding nanoemulsions are added to the overall particle size, which can generate substantial increases in particle size.^80^ Furthermore, when particles move in liquid media, electrical dipole layers of the solvent adhere to the particle surface.^81^ Therefore, the hydrodynamic diameter and the organic core, coating material, and solvents are detected.

This hydration layer is not detected in transmission electron microscopy; therefore, we obtain information only from the organic core. Inconsistencies between DLS and electron microscopy analyses were also observed in other studies.^82^

### Freeze–Thaw Cycle and Heating–Cooling Cycle

**Figure 8:**
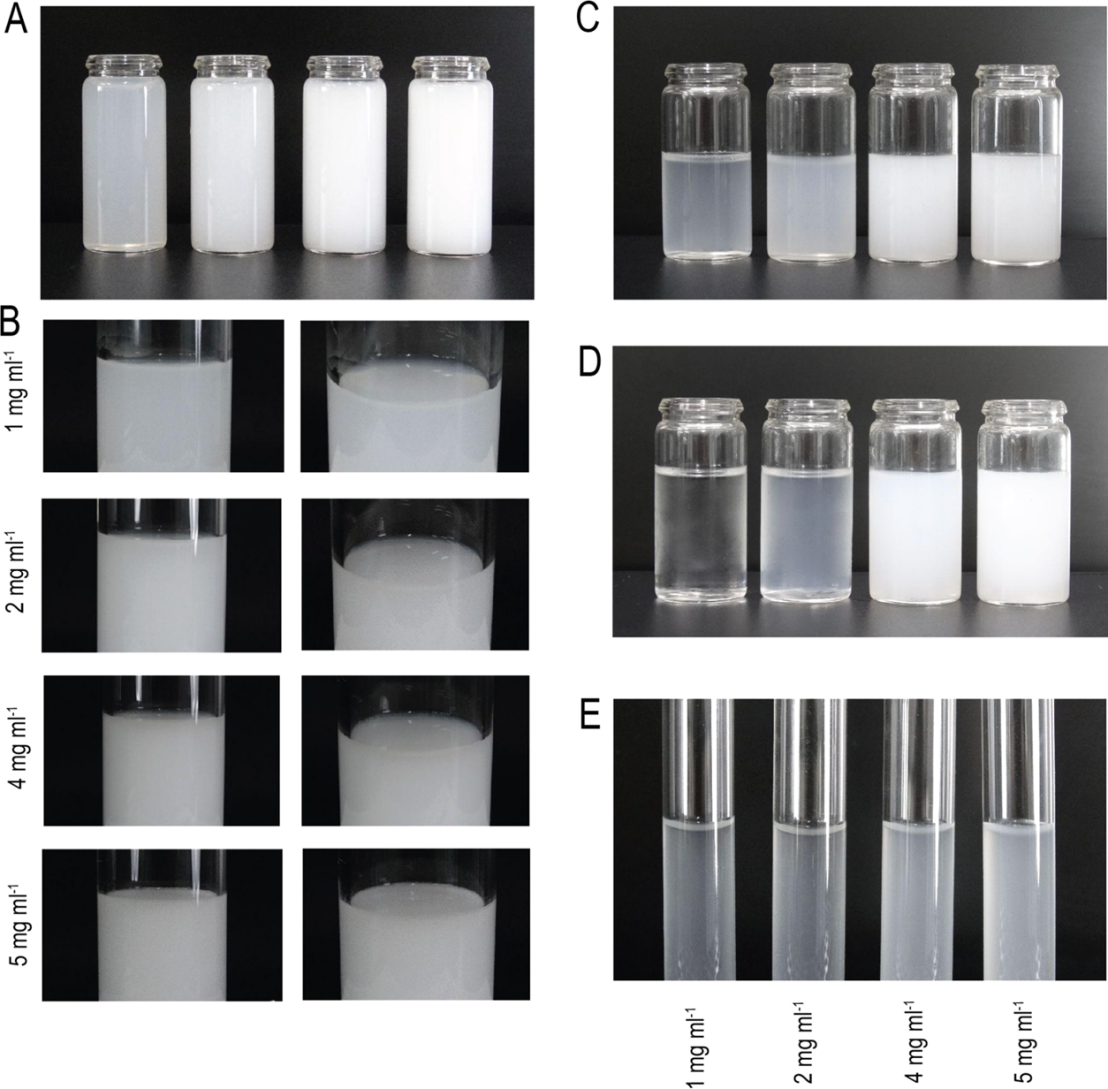
Nanoemulsions of Copaiba balsam oil at 1, 2, 4, and 5 mg mL^–1^ after formulation from right to left, respectively (A) and evaluations of creaming and cracking (B), Freeze–thaw cycle (C), heating–cooling cycle (D), and the macroscopic appearance after dilution (E).

Accelerated stability was assessed using a heating–cooling cycle. Before testing, the nanoemulsions were photographed after being formulated and used as a comparative standard (Figure 8A). The formulations were subjected to a freeze–thaw cycle, and it was observed that the nanoemulsions did not show phase separation and creaming formation after three successive freeze–thaw cycles. However, the 1 and 2 mg mL^–1^ nanoemulsions showed slight macroscopic modification, unchanged at 4 and 5 mg mL^–1^ concentrations (Figure 8C). The nanoemulsions at concentrations of 4 and 5 mg mL^–1^ showed good physical stability. In the heating–cooling cycle, the 1 and 2 mg mL^–1^ nanoemulsions showed alterations, where there was total separation and a blue aspect of the formulation, respectively (Figure 8D).

After consecutive freeze–thaw cycles in the 1 and 2 mg mL^–1^ formulations, the altered macroscopic appearance may be related to possible destabilization or decrease in particle size. During the freezing and thawing process, crystallization may be generated, causing the breakage of the surface film that the surfactant promotes around the particles, generating coalescence and possible separation of the immiscible phases (oil and water).^26^ High temperatures can influence the destabilization process of nanoemulsions, as they reduce the oil’s viscosity, thus increasing collisions between particles and the difference in density between phases. Furthermore, tween 80, a nonionic surfactant, decreases its relative solubility at high temperatures.^83,84^ However, in the heating–cooling cycle, the 2 mg mL^–1^ nanoemulsion acquired a bluish appearance, which resembles macroscopically the characteristics of smaller particles in microemulsions. The phenomenon of particle reduction over storage time was also observed in nanoemulsions containing lemon oil, which became transparent after 15 and 30 days.^63^

### Creaming and Cracking

Cracking refers to the total separation of oil and water into two phases. However, creaming does not help separate immiscible liquids and can be reconstituted by shaking or mixing, which does not occur with cracking.^85^ The nanoemulsions did not show the formation of creaming or cracking after 1 day of formulation (Figure 8B).

### Dilution Test and Transmittance Measurement

The nanoemulsions were diluted, and optimized nanoemulsions (o/w) were observed and evaluated for posteluted phase inversion. Nanoemulsions of 1, 2, 4, and 5 mg mL^–1^ showed no sign of phase inversion, precipitation, or separation. The observed findings confirm that the nanoformulations are stable (Figure 8E). Nanoemulsions of 1, 2, 4, and 5 mg mL^–1^ obtained transmittance percentages of 99.7 ± 0.049, 99.7 ± 0.023, 99.2 ± 0.018, and 99.1 ± 0.027%. Transmittance values close to 100% indicate that the nanoformulations were clear when diluted.^31^

The solubilization of the nanoemulsion in water demonstrates the system’s compatibility with aqueous fluids, allowing it to be diluted for administration purposes without problems of phase inversion or precipitation and destabilization.

### Macroscopic Stability

**Figure 9:**
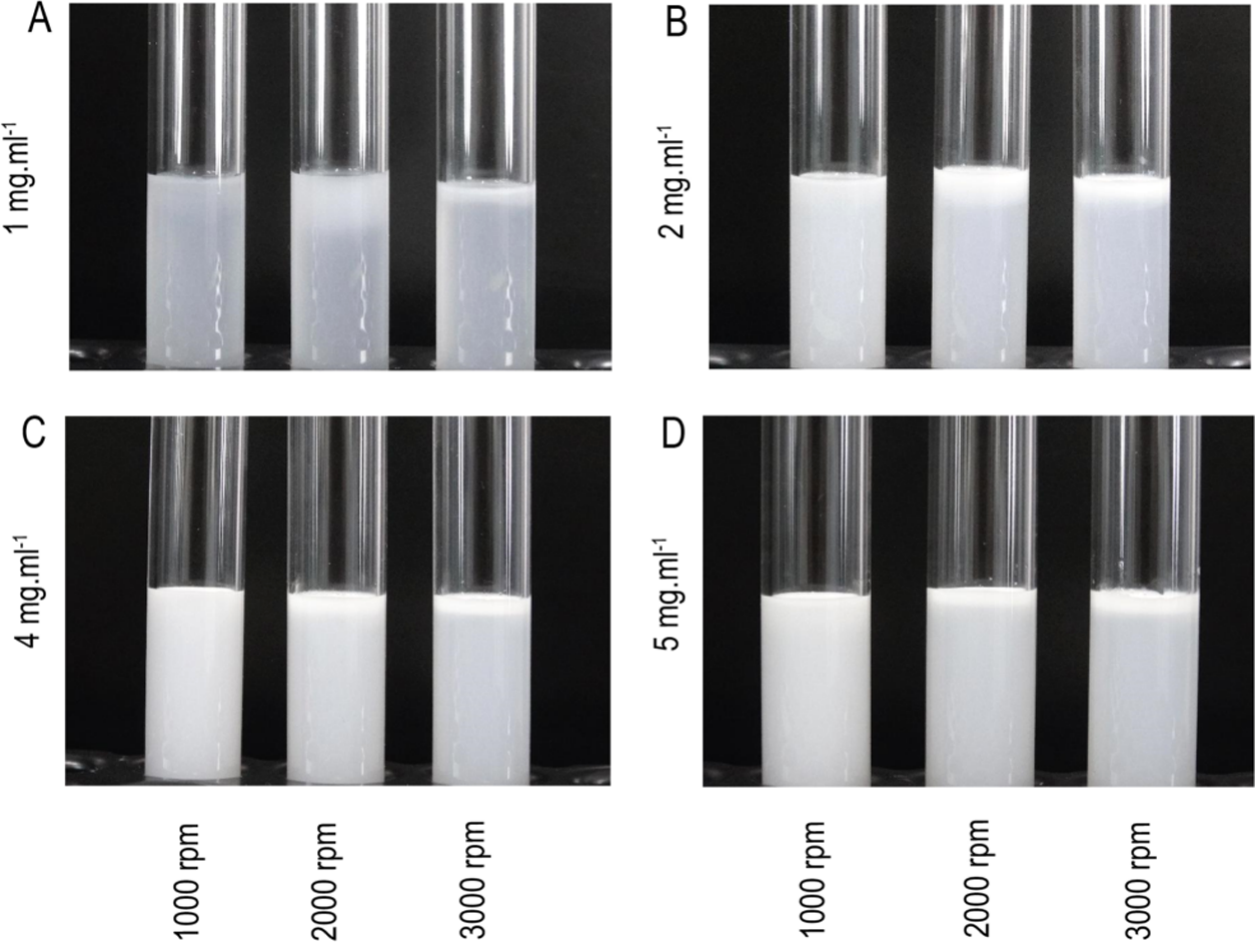
Nanoemulsion of Copaiba balsam oi1 at 1 (A), 2 (B), 4 (C), and 5 mg mL^–1^ (D) after intense stress generated by centrifugal force.

After intense stress generated by centrifugal force, the nanoemulsions were evaluated for their degree of macroscopic stability. At concentrations of 1, 2, 4, and 5 mg mL^–1^, phase separation, creaming, and flocculation were not observed after centrifugation at 1000 rpm considered stable under this stress (Table 3). However, the presence of creaming was detected after 2000 and 3000 rpm; despite the appearance of creaming, the nanoemulsions did not show signs of phase separation, flocculation, and precipitation even when subjected to intense stress. When mixing the centrifuged nanoformulation, the surface creaming film disappeared (Figure 9).

**Table 3:** Phase Separation, Creaming, and Flocculation of Nanoemulsions after Centrifugation^a^

	stirring speed (rpm)	phase separation	creaming	creaming (mm)	flocculation
1 mg mL^–1^	1000	not detected	not detected		not detected
	2000	not detected	detected	1.92 ± 0.01	not detected
	3000	not detected	detected	2.57 ± 0.004	not detected
2 mg mL^–1^	1000	not detected	not detected		not detected
	2000	not detected	detected	2.11 ± 0.18	not detected
	3000	not detected	detected	2.76 ± 0.007	not detected
4 mg mL^–1^	1000	not detected	not detected		not detected
	2000	not detected	detected	0.02 ± 0.005	not detected
	3000	not detected	detected	3.77 ± 0.01	not detected
5 mg mL^–1^	1000	not detected	not detected		not detected
	2000	not detected	detected	2.1 ± 0.1	not detected
	3000	not detected	detected	3.65 ± 0.02	not detected

a The degree of creaming was measured in triplicate, calculated to mean ± SD, and expressed in mm.

Generally, applying centrifugal forces to nanoemulsions results in instability phenomena such as precipitation, phase separation, creaming, and particle size increase.^84^ Nanoemulsions due to thermodynamic instability likely exhibit creaming/phase separation in long-term storage. The free energy of colloidal dispersion is greater than the free energy of the separated phase, determining the thermodynamic instability of the nanoemulsion.^25^ Furthermore, the stability of the nanoemulsion is directly proportional to the gravitational force. The centrifugation test can accelerate the destabilization process by stimulating the aging of formulations.^86^

Therefore, our nanoemulsions are stable when subjected to stress at 1000 rpm, however, we believe that the low levels of surfactant in the nanoformulations favored nonresistance to stress at 2000 and 3000 rpm.

### Copaiba Balsam Oil Nanoemulsion Caused Minor Toxicity in the *C. elegans* Model

**Figure 10:**
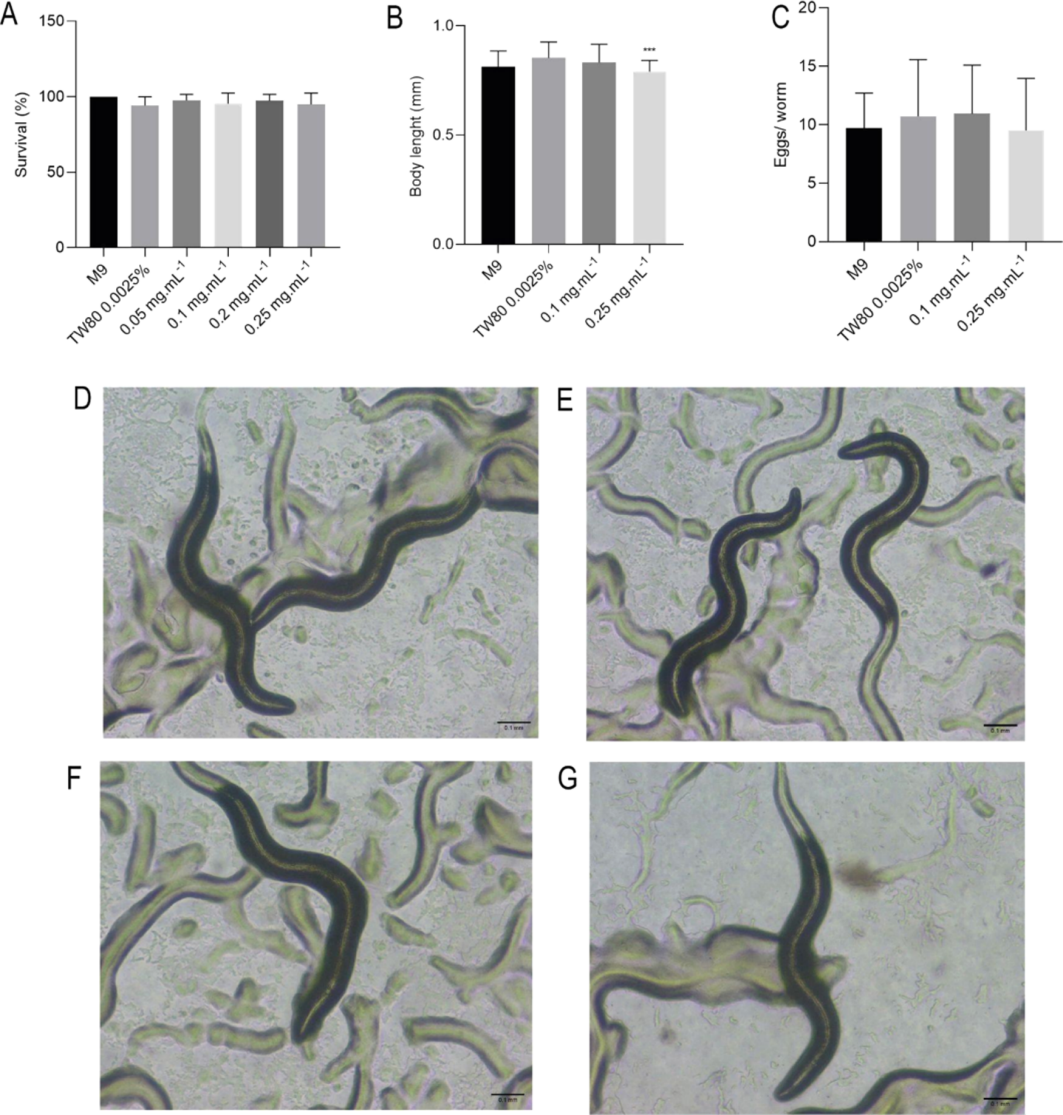
*C. elegans* survival (A), body length (B), egg production (C) and ∼42 h (L4 larval stage) after exposition to M9 buffer (D), tween 80 (E), 0.1 mg mL^–1^ (F), and 0.25 mg mL^–1^ (G) in NGM plate seeded with *E. coli*. The scale bars are represented in 0.1 mm. In the behavior assay, the animals (wild-type, N2) were maintained at 20 °C and scored 42 h after exposure. Data are represented as mean ± SD. Statistical significance was determined by One-way ANOVA followed by Bonferroni’s test. ***p < 0.001.

*C. elegans* was exposed to the nanoemulsion in the L1 larval stage and observed for 24 h. The expositions were realized from the stock nanoemulsions of 2 and 5 mg mL^–1^ to reach the final concentrations of 0.1 and 0.25 mg mL^–1^ in NGM plates. The animals did not show significant differences in survival rate compared to controls (Figure 10A).

The survival assay using *C. elegans* to evaluate the toxicity of *Copaifera sp.* has been successfully implemented in other studies, as it can provide a low-cost in vivo experiment model similar to the human metabolic pathways.^87^ This study used the compound ent-polyalthic acid from *Copaifera lens* extract at concentrations, and the results showed that LC50 was determined at 1000 μg mL^–1^ after 48 h of incubation. Thus, at concentrations below 1000 μg mL^–1^, the compound was nontoxic to the nematode.

Other researchers also observed that extracts from leaves of *Copaifera reticulata* and *Copaifera paupera* did not affect the survival of animals exposed to the compound. However, *C. reticulata* and *C. paupera* extracts were able to increase survival rates by 68 and 75% against fungal infection, respectively.^88^ Our finding allows us to observe that nanoemulsions of Copaiba balsam oil cannot generate toxicity that affects animal survival capacity and viability.

A body length assay is an exquisite way of analyzing the worms’ development and its regulation after nanoemulsion exposure.^89^ These performance results were summarized in Figure 10B, as it shows no significant length changes between the groups caused by treatment. However, animals treated with 0.25 mg mL^–1^ of nanoemulsion showed a significant reduction in body length (*p =* 0.0006). In other words, it demonstrates that the use of these nanoformulations with concentrations below is a nontoxic compound since it possibly cannot establish effects on the pathways that control the development of *C. elegans*.

The egg production performance allowed the analysis of the number of eggs present in the adult animals’ uterus. In egg production, the nanoemulsion could not change the number of eggs produced by the animal (adult day 1, ∼72 h after the treatment) after exposure, showing that the nanoformulation is incapable of generating reproductive toxicity at tested concentrations (Figure 10C). Figure 10D–G show *C. elegans* at the L4 larval stage after ∼44 h of exposure to the vehicle and nanoemulsions.

**Figure 11:**
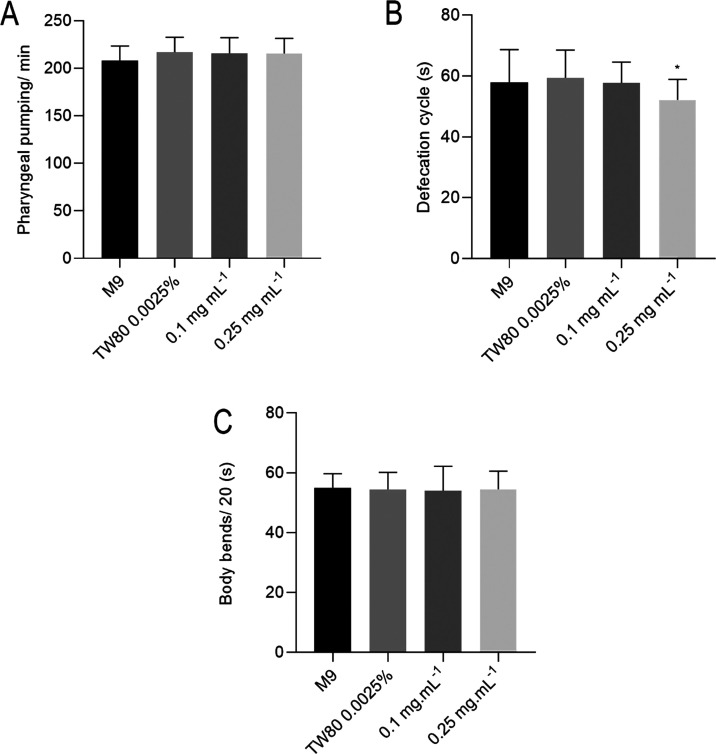
*C. elegans* behaviors. Pharyngeal pumping (A), defecation cycle (B), and body bends (C). Statistical significance was determined by One-way ANOVA followed by Bonferroni’s test. *p < 0.05.

Behavioral activities, such as movement, pharyngeal pumping, social behavior, and defecation cycle, result in mechanisms that control the metabolic activity of *C. elegans*.^90^ The behavior of *C. elegans* is an essential tool being used in the assessment of chemical toxicity, including pesticides and solvents.^91^ Thus, we evaluated the animal’s behavior after exposure to the nanoemulsion. No absence or reduction of pharyngeal pumping was observed in worms exposed to nanoemulsion compared to the control group (Figure 11A). The peristaltic movement of contraction of the animal’s gastrointestinal tract was also not altered compared to the control group. However, animals exposed to 0.25 mg mL^–1^ of nanoemulsion showed a significant reduction in defecation when compared to the solvent (*p* < 0.05) (Figure 11B).

Pharyngeal pumping is an essential assay to assess the feeding behavior of the worm by analyzing food intake.^92^ In this study, as represented in Figure 11A, the nanoemulsion did not show significant differences when compared to the control, which suggests that the food ingestion was not affected by the nanoemulsion.

Defecation cycle assay is an important marker of toxicity in *C. elegans* digestive tube.^93^ As we showed in Figure 11B, the formulation did not induce significant differences in most of the treated groups; nevertheless, the major concentration used induced a significant decrease in defecation cycle time, probably due to the increase in the number of nanoparticles in the medium. As the defecation cycle reduces, the number of defecations is faster. The defecation cycle is controlled by acetylcholine levels in synaptic clefts. However, levels of Ca^2+^ and the neurotransmitter γ-aminobutyric acid also regulate this behavior.^94^ The changes in the defecation cycle observed may indicate possible influences of the nanoemulsion’s chemical constituents on behavior by these systems. Furthermore, caryophyllene acts on cannabinoid receptors, which in *C. elegans* are found mainly in the intestine and regulate intestinal motility.^95−97^ Another researcher observed that activation of cannabinoid CB1 receptors increased the number of intestinal contractions, reducing the defecation interval.^94^

*C. elegans* has distinct forms of locomotion, such as swimming and crawling,^98^ that can be affected by exposure to different substances. The results showed that the nanoparticles did not impact the number of body bends and did not indicate toxicity in worms’ body movement (Figure 11C).

In conclusion, our nanoformulation did not show toxicity at the tested concentrations because it was not capable of altering significantly the worm’s behaviors. Our results corroborate previous findings; since Copaiba balsam oil is used in traditional medicine and even changing the formulation, low toxicity remains.^99^ It is well-known that toxic substances induce behavioral interferences and that is one of the main reasons *C. elegans* is a good tool for toxicology assessment.^100^ The compound also did not alter the animal’s survival and normal development, which corroborates the use of Copaiba balsam oil by humans over the years without causing toxic effects.^10,101^ Considering these parameters, our results indicate positive perspectives for the Copaiba balsam oil nanoemulsion, once its high stability and low toxicity in *C. elegans* promote further research focused on discovering its pharmacological mechanisms and applications. It is known that oxidative stress levels, for example, are an important indicator involved in cancer, but also in cardiovascular and degenerative diseases.^102^ Therefore, compounds that have the ability to perform as an antioxidant have been the focus of pharmacological research for a long time. Thus, as of these initial results, Copaiba balsam oil nanoemulsion potentials on oxidative stress and other disease-related processes need to be evaluated in *C. elegans*, a useful pharmacological model system that allows the analysis of important pathways.^103^

## Conclusions

Nanoemulsions with Copaiba balsam oil in low surfactant concentrations were synthesized and evaluated for stability. Our study produced the first nanoemulsion of Copaiba balsam oil with low levels of surfactant, whose higher concentrations of Copaiba balsam oil showed better physicochemical and thermodynamic stabilities, produced smaller particle size, and the nanoformulations remained polydisperse for one and two years. Once subjected to different pH, the size remained as expected, with an increase observed at the lowest concentrations, without changes in PdI, and negative electrical charges were reduced, which may indicate appropriate stability for different routes of administration. β-caryophyllene was the majority constituent of both Copaiba balsam oil and nanoemulsion, indicating the stability of the molecular profile during the nanoformulation process. Tests in the nonvertebrate animal model revealed favorable indications of nanoemulsions on the physiological state, given the minor toxicity observed, which corroborates the wide use of Copaiba balsam oil in traditional medicine. Altogether, the stability and toxicity results support the use of nanoemulsions in future studies that consider enhancing pharmacological utilization in complex experimental evaluations.

## Supporting Information Available

Chromatogram of Copaiba balsam oil; chromatogram of the nanoemulsion; transmission electron microscopy photomicrographs; pH and viscosity table (PDF)
