# Supplementary material for: Synthesis and Physicochemical Stability of a Copaiba Balsam Oil (Copaifera sp.) Nanoemulsion and Prospecting of Toxicological Effects on the Nematode Caenorhabditis elegans
Source: ACS Omega. 2024 Sep 6;9(37):39100–18. doi: 10.1021/acsomega.4c05930 (PMC11411554; doi:10.1021/acsomega.4c05930)
Supplement: Supplementary file 1 — ao4c05930_si_001.pdf [file ao4c05930_si_001.pdf]

Supplementary Informations

Synthesis and physicochemical stability of a Copaiba balsam oil (*Copaifera sp.*) nanoemulsion and prospecting of toxicological effects on the nematode *C. elegans*

Iverson Conrado Bezerra[1], Emily Raphaely Souza dos Santos[1], Jocelin Santa Rita Bisneto[1], Paloma Paschoal Perruci[1], Angela Iasmin de Barros Ferreira[1], Daniel Charles dos Santos Macêdo[1,3], Mateus Araújo da Luz[4], Taynah Pereira Galdino[4], Giovanna Machado[2], Nereide Stela Santos Magalhães[1,3], Mariane Cajuba de Britto Lira Nogueira[1], Priscila Gubert[1,5*]

Affiliations:

[1] Keizo Asami Institute (iLIKA), Federal University of Pernambuco, Recife, Brazil.

[2] Northeast Strategic Technologies Center (CETENE), Recife, Brazil.

[3] Department of Pharmaceutical Sciences, Federal University of Pernambuco, Recife, Brazil.

[4] Northeast Biomaterials Assessment and Development Laboratory (CERTBIO), Federal University of Campina Grande, Campina Grande, Brazil.

[5] Federal University of Western Bahia (UFOB), Barreiras, Brazil.

* Corresponding Author's Email: prikagubert@gmail.com

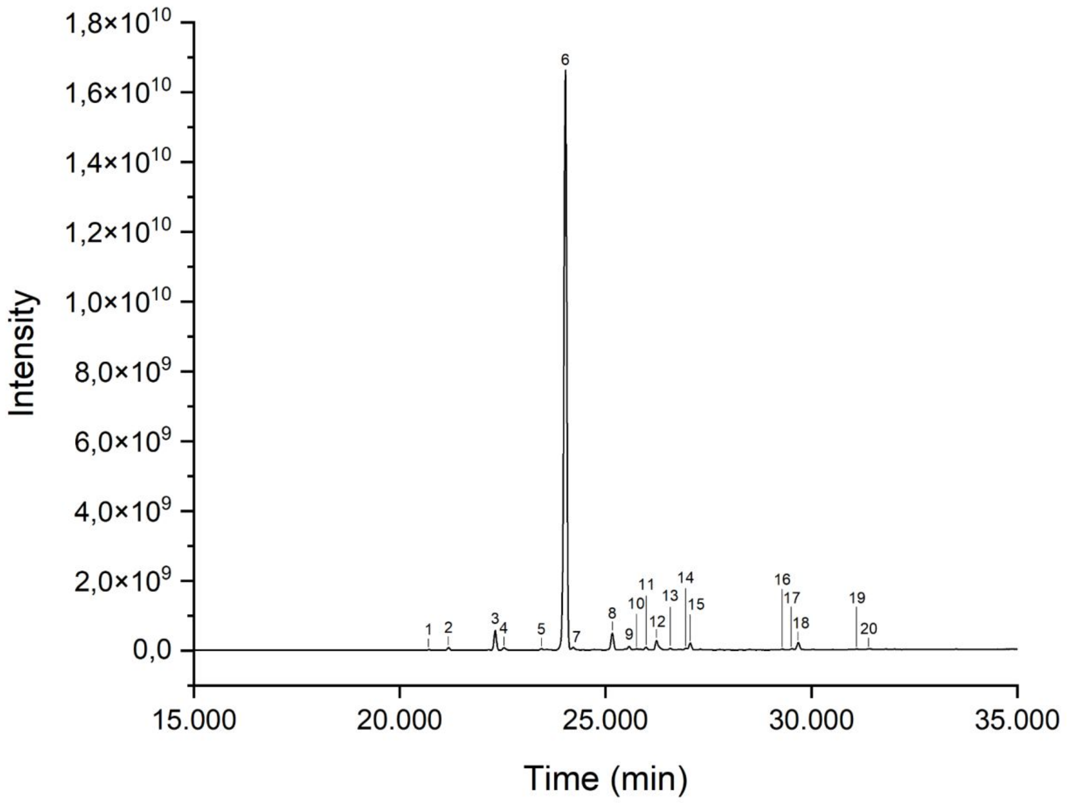

Figure S1: Chromatogram of Copaiba balsam oil in GC-MS. The number and names of compounds are represented in table 1.

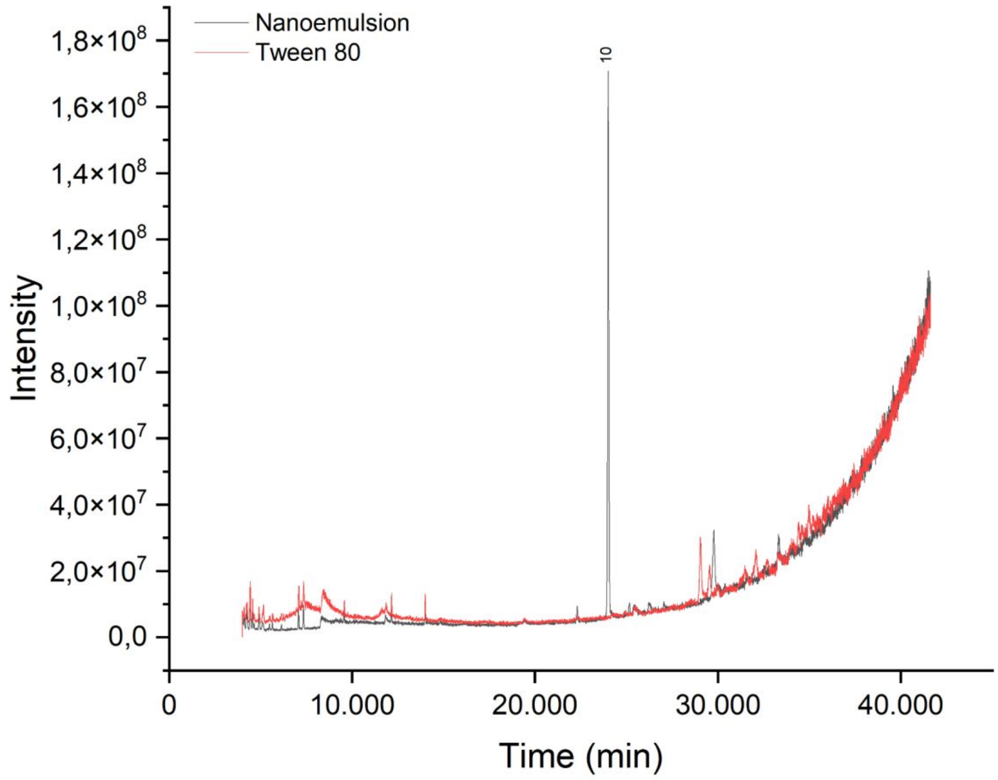

Figure S2: Chromatogram of nanoemulsion of Copaiba balsam oil in GC-MS.

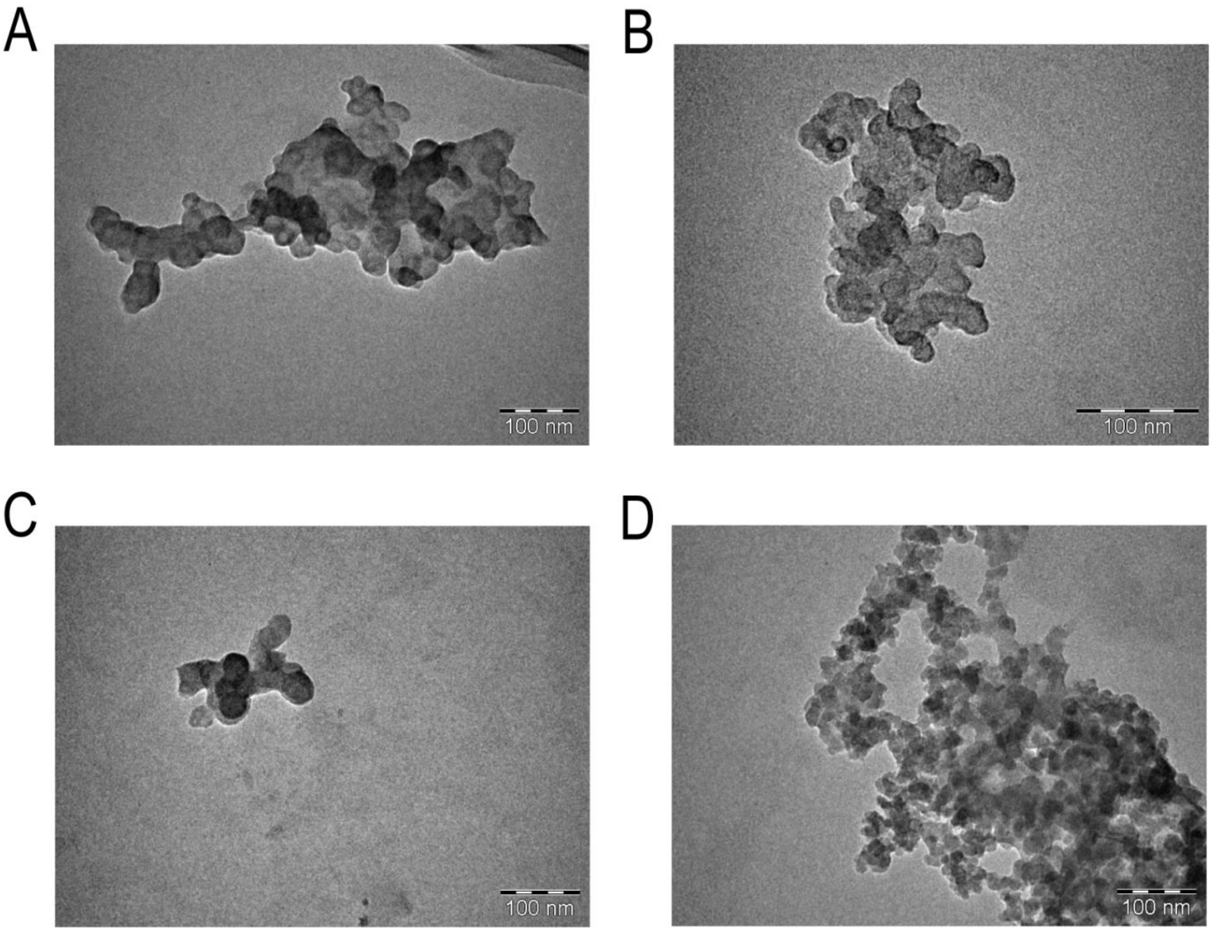

Figure S3: Transmission electron microscope micrograph of 2 (A) and 5 $mg.ml^{-1}$ (B, C, and D) nanoemulsions.

Table S1: pH and viscosity of nanoemulsions at 25 °C. Datas are represented in Mean±SD.

	1 $mg.ml^{-1}$	2 $mg.ml^{-1}$	4 $mg.ml^{-1}$	5 $mg.ml^{-1}$
Viscosity (mPas.s)	1.14 ± 0.05	1.14 ± 0.05	1.14 ± 0.04	1.14
pH	5.72 ± 0.4	5.85 ± 0.04	5.85	5.88 ± 0.04
